# PDBe-KB: collaboratively defining the biological context of structural data

**DOI:** 10.1093/nar/gkab988

**Published:** 2021-11-10

**Authors:** PDBe-KB consortium, Mihaly Varadi, Stephen Anyango, David Armstrong, John Berrisford, Preeti Choudhary, Mandar Deshpande, Nurul Nadzirin, Sreenath S Nair, Lukas Pravda, Ahsan Tanweer, Bissan Al-Lazikani, Claudia Andreini, Geoffrey J Barton, David Bednar, Karel Berka, Tom Blundell, Kelly P Brock, Jose Maria Carazo, Jiri Damborsky, Alessia David, Sucharita Dey, Roland Dunbrack, Juan Fernandez Recio, Franca Fraternali, Toby Gibson, Manuela Helmer-Citterich, David Hoksza, Thomas Hopf, David Jakubec, Natarajan Kannan, Radoslav Krivak, Manjeet Kumar, Emmanuel D Levy, Nir London, Jose Ramon Macias, Madhusudhan M Srivatsan, Debora S Marks, Lennart Martens, Stuart A McGowan, Jake E McGreig, Vivek Modi, R Gonzalo Parra, Gerardo Pepe, Damiano Piovesan, Jaime Prilusky, Valeria Putignano, Leandro G Radusky, Pathmanaban Ramasamy, Atilio O Rausch, Nathalie Reuter, Luis A Rodriguez, Nathan J Rollins, Antonio Rosato, Paweł Rubach, Luis Serrano, Gulzar Singh, Petr Skoda, Carlos Oscar S Sorzano, Jan Stourac, Joanna I Sulkowska, Radka Svobodova, Natalia Tichshenko, Silvio C E Tosatto, Wim Vranken, Mark N Wass, Dandan Xue, Daniel Zaidman, Janet Thornton, Michael Sternberg, Christine Orengo, Sameer Velankar

## Abstract

The Protein Data Bank in Europe – Knowledge Base (PDBe-KB, https://pdbe-kb.org) is an open collaboration between world-leading specialist data resources contributing functional and biophysical annotations derived from or relevant to the Protein Data Bank (PDB). The goal of PDBe-KB is to place macromolecular structure data in their biological context by developing standardised data exchange formats and integrating functional annotations from the contributing partner resources into a knowledge graph that can provide valuable biological insights. Since we described PDBe-KB in 2019, there have been significant improvements in the variety of available annotation data sets and user functionality. Here, we provide an overview of the consortium, highlighting the addition of annotations such as predicted covalent binders, phosphorylation sites, effects of mutations on the protein structure and energetic local frustration. In addition, we describe a library of reusable web-based visualisation components and introduce new features such as a bulk download data service and a novel superposition service that generates clusters of superposed protein chains weekly for the whole PDB archive.

## INTRODUCTION

The structure of biological macromolecules and their complexes is invaluable for understanding their functions (1,2). These structures allow researchers to infer atomic-level mechanisms of biological systems and enable them to modulate biological processes through, for example, structure-based drug design, synthetic biology, and protein engineering (3–5).

For 50 years, the Protein Data Bank (PDB), managed by the worldwide Protein Data Bank consortium (wwPDB) (6), has served as the global archive for experimentally determined structures. To date, the PDB contains over 180 000 structures of 55 000 distinct proteins, with around 12 000 new PDB entries deposited annually (7). Advances in structure determination promise that the repertoire of known structures will continue to grow, mainly owing to the widespread application of single-particle cryo-electron microscopy yielding high-resolution structures. Nevertheless, the known sequence space is expanding even faster (8); only 0.27% of protein sequences in the Universal Protein Data Resource (UniProt) has structural representations in the PDB. This gap between the knowledge of sequences and structures will continue to grow (6,9–11). Although high-accuracy predicted models made public recently have the potential to expand the structural coverage of the sequence space massively, these methods still have limitations in modelling mutant structures and assemblies (12–14).

While macromolecular structures are invaluable, they often need to be interpreted using additional structural and functional annotation layers to answer specific biological questions (15). For example, annotating structures with druggable surface pockets, molecular channels or identifying residues critical for stabilising an interaction interface can give more in-depth insights than 3D coordinates alone (16–18).

Many specialist data resources, and scientific software provide such annotations, and their number keeps growing (15). However, while having access to such a rich ecosystem of annotations empowers the scientific community, it is becoming increasingly difficult to track and combine these data. While most annotations are openly accessible, they may not be easily findable, and the lack of standard data formats often hinders interoperability and reusability.

We established PDBe-KB in 2018 to make these annotations FAIR (i.e. findable, accessible, interoperable, reusable) through a global collaboration between PDBe and leading specialist data providers and scientific software developers (15). This collaborative consortium aims to place macromolecular structures in their biological context by providing FAIR access to structural, functional and biophysical annotations of protein, nucleic acid and small-molecule structures in the PDB.

PDBe-KB is an open consortium transparently governed by a collaboration guideline (https://pdbe-kb.org/guidelines). Contributing data resources are requested to provide their PDB residue or PDB chain annotations in a data format defined and maintained by the consortium (https://github.com/PDBe-KB/funpdbe-schema). This data exchange format evolves according to the partner resources' requirements, and the consortium reviews the specification during annual PDBe-KB workshops. In addition, PDBe-KB makes the integrated annotations openly accessible to the scientific community through file transfer protocol (ftp://ftp.ebi.ac.uk/pub/databases/pdbe-kb), programmatic access (https://pdbe-kb.org/graph-api) and web pages (https://pdbe-kb.org/proteins). As a result, the consortium grew from 18 to 30 collaborating data resources from 11 different countries in the past two years (Table 1).

**Table 1.** Data resources and scientific software contributing annotations to PDBe-KB

Partner resource	Resource leader	Type of annotations	Country
14–3–3-Pred (19)	G. Barton	Binding site predictions	GBR
3D Complex (20)	E. D. Levy, S. Dey	Interaction interfaces	ISR
3DLigandSite (21)	M. Wass	Binding site predictions	GBR
AKID (22)	M. Helmer-Citterich	Kinase-target predictor	ITA
Arpeggio (23)	T. Blundell	Ligand interactions	GBP
CamKinet *(in preparation)*	M. Kumar	Curated post-translational modification sites	DEU
canSAR (16)	B. al-Lazikani	Druggable pocket predictions	GBR
CATH-FunSites (24)	C. Orengo	Functional site predictions	GBR
ChannelsDB (18)	R. Svobodova, K.Berka	Molecular channels	CZE
COSPI-Depth (25)	M. S. Madhusudhan	Residue depth	IND
Covalentizer (26) (new)	N. London	Predicted covalent binding molecules	ISR
DynaMine (27)	W. Vranken	Backbone flexibility predictions	BEL
ELM (28)	T. Gibson	Short linear motifs	DEU
EMV (29) (new)	J. R. Macias	EM validation annotations from 3DBionotes	ESP
EVcouplings (30) (new)	D. Marks	Covariations	USA
FireProt DB (31) (new)	J. Damborsky	Effects of mutations on protein stabilities	CZE
FoldX (32)	L. Serrano	Energetic consequences of mutations	ESP
FrustratometeR (33)(new)	R. Gonzalo Parra	Energetic local frustration	ESP
KinCore (34) (new)	R. Dunbrack	Conformational annotations	USA
KnotProt (35) (new)	J. Sulkowska	Topology annotations	POL
M-CSA (36)	J. Thornton	Curated catalytic sites	GBR
MetalPDB (37)	C. Andreini, A. Rosato	Curated metal-binding sites	ITA
Missense3D (38)	M. Sternberg	Mutations in human proteome	GBR
MobiDB (39) (new)	S. Tosatto	Consensus disorder predictions	ITA
P2rank (40)	D. Hoksza	Binding site predictions	CZE
POPS (41)	F. Fraternali	Solvent accessibility	GBR
ProKinO (42)	N. Kannan	Curated post-translational modification sites	USA
Scop3P (43) (new)	L. Martens, W. Vranken	Phosphorylation sites	BEL
SKEMPI (44) (new)	J. Fernandez-Recio	Thermodynamic effects of mutations	ESP
WEBnm@ (45) (new)	N. Reuter	Flexibility predictions	NOR

PDBe-KB integrates annotations from 30 partner resources who provide functional, biophysical and biochemical annotations.

## IMPLEMENTATION

**Figure 1.**
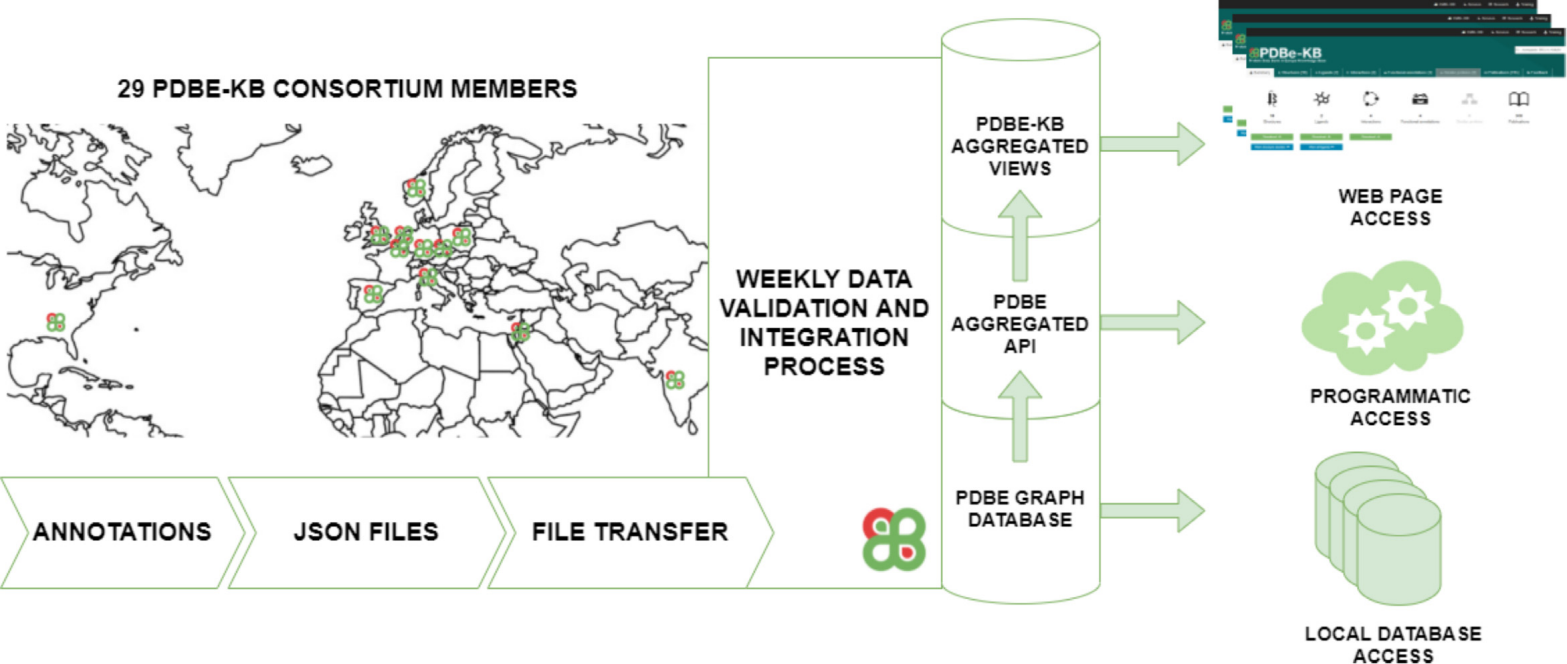
Schematic overview of the PDBe-KB infrastructure. PDBe-KB partner resources convert their annotations to a predefined JSON format and transfer these file sets via FTP. Weekly data validation and integration processes parse and load the annotations into the PDBe graph database. A rich set of API endpoints expose the data and power the PDBe-KB aggregated views. Researchers can access the data by setting up a local instance of the graph database, using the API endpoints, or visiting the aggregated view pages.

The infrastructure of PDBe-KB consists of four main components. These are (i) a deposition system for annotations; (ii) a graph database that integrates annotations with the core PDB data; (iii) a rich set of application programming interface (API) endpoints that provide access to the data; (iv) a set of reusable web components that are combined to create the PDBe-KB aggregated views (Figure 1).

### Data deposition

The data deposition system changed significantly to ensure scalability as more partner data resources joined the consortium. Data providers are required to convert their annotations to JavaScript Object Notation (JSON) files, according to the data exchange format specification, which is available at https://github.com/PDBe-KB/funpdbe-schema. Collaborators then copy their JSON files to private FTP areas provided by PDBe-KB, hosted at EMBL-EBI in Hinxton. A weekly running data processing pipeline parses, validates and integrates the data from these JSON files into the PDBe graph database. When displaying or providing access to annotations from any PDBe-KB partner resources, we provide direct links the users can follow to find the original data set from the corresponding database or scientific software.

### Data access

The PDBe graph database is an up-to-date knowledge graph that contains the latest PDB data, linked to the corresponding UniProt accessions and integrated with structural, functional and biophysical annotations. It is implemented in Neo4j v3.5 and has over 1 billion nodes and 1.5 billion edges. The database is openly accessible at https://pdbe-kb.org/graph-download, and users can install it in-house to use it as a research tool for data mining. It requires ∼0.5TB of local storage space, preferably on an SSD drive with a recommended 6 GB RAM and eight cores.

The PDBe aggregated API provides programmatic access to all the aspects of the data contained within the graph database. As we integrated new annotations into PDBe-KB, we have expanded the API and currently provide over 90 different API endpoints. We have described these endpoints and provided use case examples elsewhere (46). The API is available at https://pdbe-kb.org/graph-api.

### Web components library

PDBe-KB web pages use modular web components which can be reused and customised easily. We have created an open-source library for these components so that data service developers can use them as plugins for visualising structural data. In addition, they provide built-in support for the PDBe aggregated API, allowing developers to display data from PDBe-KB conveniently. We implemented the web components using the AngularJS framework. They are available and freely reusable from GitHub at https://github.com/PDBe-KB?q=component.

### New features on the aggregated views of proteins

We continuously develop the PDBe-KB pages, displaying all the available structural information for a protein, keyed on a UniProt accession. We call these pages aggregated views of proteins. In addition, we have added several features, in particular: (i) a superposition service to visualise protein chains clustered by structural similarity; (ii) a bulk download service that provides easy access to all the coordinate files, validation reports, sequences for a protein of interest; (iii) a section dedicated to processed proteins and (iv) annotations for small molecules and macromolecular interaction partners.

**Figure 2.**
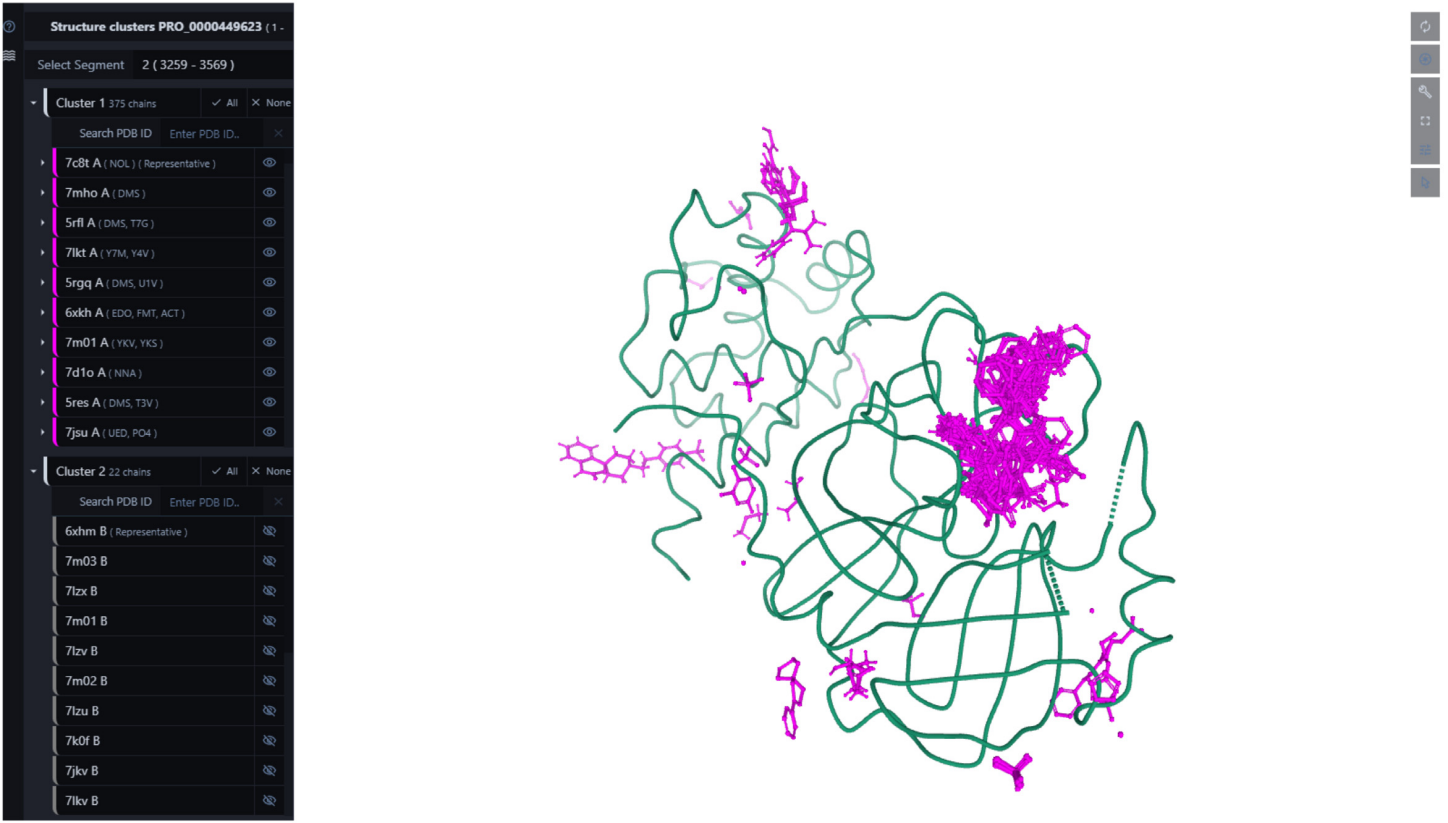
Superposition of protein chains and ligand molecules. The aggregated views of proteins provide access to superposed protein segments and offer a display mode that overlays all the observed ligands on representative chains from superposition clusters. The figure displays the ligand superposition of all the available small molecules in PDB structures of the 3C-like proteinase nsp5 of SARS-CoV-2.

We have designed a weekly process to generate superposed UniProt segments for the whole PDB archive. We described the details of the data process on the public Wiki pages of PDBe-KB at https://github.com/PDBe-KB/pdbe-kb-manual/wiki/Superposition. The superposed coordinates are made available on the aggregated views of proteins, where clustered, superposed PDB chains can be displayed using the interactive 3D molecular viewer, Mol* (47), by clicking on the ‘view structure clusters’ buttons. We also provide a unique superposition view that displays all the ligand molecules overlaid on representative chains from superposition clusters (Figure 2). In the example below, we display the 3C-like proteinase nsp5 of SARS-CoV-2. By overlaying all the bound small molecules, researchers can identify a frequently populated binding pocket.

**Figure 3.**
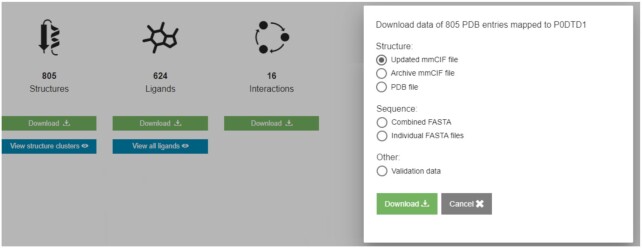
Bulk data download service. The aggregated views of proteins provide a graphical user interface to a new bulk data download service which enables researchers to download all the coordinates, sequences and validation data available for a protein of interest.

Previously, it was cumbersome to download all the structural and functional data available for a protein of interest from its aggregated view. We have recently designed a download service that has a graphical user interface to enable users to download coordinates (archive mmCIF, updated mmCIF and PDB format), sequences (FASTA format) and validation data (Figure 3). The updated mmCIF is based on the archive mmCIF file. Both files follow the same PDBx/mmCIF dictionary. The updated mmCIF has two major differences from the archive mmCIF file: (i) selected data values are cleaned up to standardise the enumerations; (ii) additional data categories and items are added as required to support PDBe data out activities and external users. An example of a standardised enumeration is the values in *_exptl.method* which is standardised and changed from uppercase to title case. Another example of additional categories is the *_chem_comp_bond* which defines the expected bond order for every bond in every component in the PDB entry. Users can download these data for all the PDB entries for a protein of interest or only those containing small molecules or macromolecular complexes. Users can also interact with the download service programmatically through a set of API endpoints. Documentation of this API is available at https://www.ebi.ac.uk/pdbe/download/api/docs.

**Figure 4.**
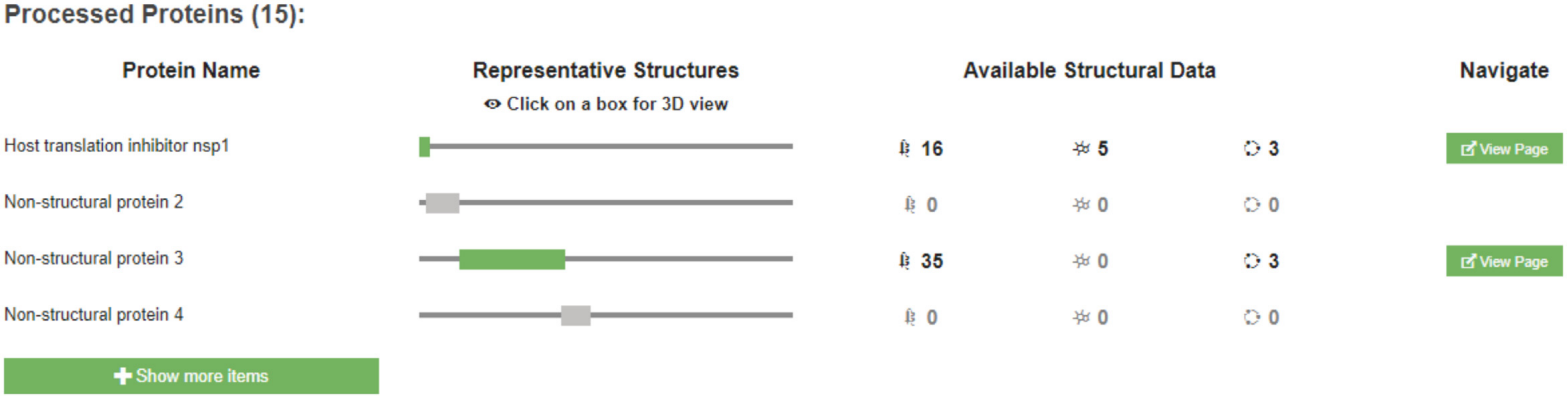
Processed proteins section. The aggregated views of proteins now include a section highlighting all the mature, processed proteins for a polyprotein. In addition, users can click on the green boxes to view the 3D structures using Mol*, and they can navigate to dedicated processed proteins pages by clicking on the ‘view page’ button.

In response to the COVID-19 pandemic, we developed a unique set of web pages focused on the proteins of SARS-CoV-2 in early 2020. However, it became apparent that we could improve the display of polyproteins. In particular, the aggregated views were not highlighting the mature, processed proteins, and users could not zoom in on these proteins. To address this, we have integrated information on processed proteins from UniProt and have added a new section that highlights the segments they occupy on the full-length polyprotein sequences (Figure 4). We now also enable users to view pages specifically for a particular processed/mature protein, using the PRO identifiers from UniProt. For example, https://www.ebi.ac.uk/pdbe/pdbe-kb/proteins/PRO_0000449633 is the dedicated page of the 2'-*O*-methyltransferase nsp16 of SARS-CoV-2, which is a processed protein from Replicase polyprotein 1ab (UniProt accession P0DTD1). These pages are available for all the processed/mature proteins with known structures, not only viral proteins.

**Figure 5.**
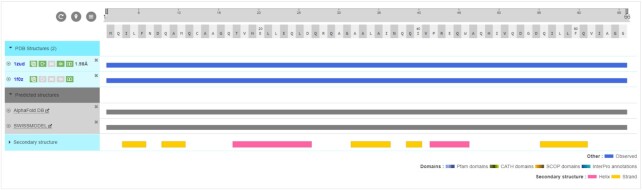
Predicted models of a protein of interest. The aggregated views of proteins now provide an overview of available predicted models from data resources such as AlphaFold DB and SWISS-MODEL.

While experimentally determining structures remains a costly and labour-intensive endeavour, there have been significant advances in the field of structural predictions. Researchers increasingly deploy Artificial Intelligence (AI) techniques to predict a protein's structure computationally from its amino-acid sequence alone (12,13,48). While the aggregated views of proteins already provided an overview of all the protein structures available in the PDB, we have expanded the scope to include predicted models from data providers such as SWISS-MODEL and AlphaFold DB (14,49) (Figure 5). Displayed in ProtVista, users can compare the structural coverage of the protein sequences and directly download predicted models.

**Figure 6.**
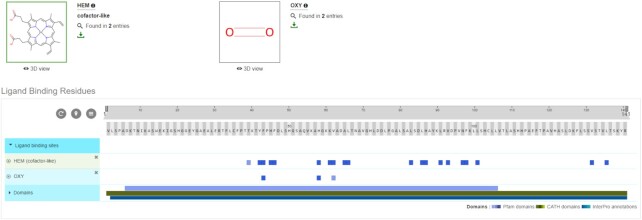
Ligand annotations. The aggregated views of proteins now display annotations for ligand molecules based on a cofactor data pipeline. Similarly, we annotate peptides and antibodies in the macromolecular interactions section.

Most of the annotations provided by the PDBe-KB partner resources focus on amino acid residues and their functions or biophysical characteristics, yet PDBe-KB has information also on molecular entities such as small molecules or macromolecular interaction partners (Figure 6). For example, using a previously developed semi-automated annotation process, we can now flag small molecules as enzyme cofactors and cofactor-like molecules (50). We display this information on the sequence feature viewer, ProtVista and ligand gallery. Similarly, we have weekly processes for identifying and annotating peptides and antibody structures, which we display in the macromolecular interactions section.

### Training and tutorials

Working together with the Training team of EMBL-EBI, we actively participated in training courses and continued to create training materials and tutorials that describe the new functionalities and changes to PDBe-KB web services and web pages. Recently, we created a set of tutorials that encompass programmatic access to PDBe data, data processing packages and web components that visualise the structures and annotations. These tutorials are available at https://pdbeurope.github.io/api-webinars/index.html.

## DISCUSSION

PDBe-KB expands the structural, functional, and biophysical annotations of molecular structure data according to its long-term goals. By allowing integrated and FAIR access to these annotations, researchers in academia and industry can take advantage of the rich ecosystem of specialist data resources and scientific software and efficiently collate data to answer specific biological questions. Since we established PDBe-KB in 2018, the collaboration grew, integrating data from 30 partner resources across 11 countries, providing over 1.2 billion residue-level annotations. Furthermore, PDBe-KB continues to be one of the main activities of the ELIXIR 3D-BioInfo community, which brings together researchers, structural bioinformatics developers and data providers to discuss and strive for data FAIRness, benefitting the broader scientific community (51).

While we plan to further improve the aggregated views of proteins, we are also developing novel aggregated web pages for ligands, providing comprehensive structural and functional information on all the observed small molecules in the PDB archive.

Finally, we would like to extend an invitation to all the data providers and scientific software developers to join the consortium and increase user exposure through this community-driven data-sharing platform and knowledge base.

In conclusion, PDBe-KB keeps evolving and makes structural data and their structural, functional, and biophysical annotations more accessible to the scientific community, reaching over 340 000 users annually either through their usage of the rich set of programmatic access endpoints or by their visits to the PDBe-KB aggregated views pages.

## DATA AVAILABILITY

PDBe-KB is available at https://pdbe-kb.org. Individual, protein-focused pages per UniProt accessions are available at https://pdbe-kb.org/proteins/P0DTD1. Documentation of the consortium members is available at https://github.com/PDBe-KB/pdbe-kb-manual/wiki. Users can download the graph database from https://pdbe-kb.org/graph-download, and users can find the aggregated API at https://pdbe-kb.org/graph-api. The PDBe-KB web component library is public at https://github.com/PDBe-KB?q=component. Finally, we make all the annotations available in JSON format from ftp://ftp.ebi.ac.uk/pub/databases/pdbe-kb.

## APPENDIX

Current PDBe-KB Consortium Members with Affiliations

Mihaly Varadi^1,*^, Stephen Anyango^1^, David Armstrong^1^, John Berrisford^1^, Preeti Choudhary^1^, Mandar Deshpande^1^, Nurul Nadzirin^1^, Sreenath S. Nair^1^, Lukas Pravda^1^, Ahsan Tanweer^1^, Bissan Al-Lazikani^2^, Claudia Andreini^3^, Geoffrey J. Barton^4^, David Bednar^5^, Karel Berka^6^, Tom Blundell^7^, Kelly P Brock^8^, Jose Maria Carazo^9^, Jiri Damborsky^5^, Alessia David^10^, Sucharita Dey^11^, Roland Dunbrack^12^, Juan Fernandez Recio^13^, Franca Fraternali^14^, Toby Gibson^15^, Manuela Helmer-Citterich^16^, David Hoksza^17^, Thomas Hopf^8^, David Jakubec^17^, Natarajan Kannan^18^, Radoslav Krivak^17^, Manjeet Kumar^15^, Emmanuel D Levy^11^, Nir London^11^, Jose Ramon Macias^9^, Madhusudhan M. Srivatsan^19^, Debora S Marks^8^, Lennart Martens^20, 21^, Stuart A McGowan^4^, Jake E McGreig^22^, Vivek Modi^12^, R. Gonzalo Parra^23^, Gerardo Pepe^16^, Damiano Piovesan^24^, Jaime Prilusky^11^, Valeria Putignano^3^, Leandro G. Radusky^25^, Pathmanaban Ramasamy^20, 21, 26^, Atilio O. Rausch^27^, Nathalie Reuter^28^, Luis A. Rodriguez^13^, Nathan J Rollins^8^, Antonio Rosato^3^, Paweł Rubach^29^, Luis Serrano^25^, Gulzar Singh^19^,Petr Skoda^17^, Carlos Oscar S. Sorzano^9^, Jan Stourac^5^, Joanna I Sulkowska^29^, Radka Svobodova^30^, Natalia Tichshenko^20, 21^, Silvio C.E. Tosatto^24^, Wim Vranken^26^, Mark N Wass^22^, Dandan Xue^28^, Daniel Zaidman^11^, Janet Thornton^1^, Michael Sternberg^10^, Christine Orengo^31^, Sameer Velankar^1*^

^1^European Molecular Biology Laboratory, European Bioinformatics Institute (EMBL-EBI), Wellcome Genome Campus, Hinxton, UK
^2^Cancer Research UK Cancer Therapeutics Unit, Division of Cancer Therapeutics, The Institute of Cancer Research, London, UK
^3^University of Florence and C.I.R.M.M.P., Magnetic Resonance Center, Sesto Fiorentino, Italy
^4^School of Life Sciences, University of Dundee, Dundee, UK
^5^Masaryk University & International Centre for Clinical Research, St. Anne's University Hospital Brno, Department of Experimental Biology and RECETOX, Brno, Czech Republic
^6^Palacky University Olomouc, Department of Physical Chemistry, Olomouc, Czech Republic
^7^University of Cambridge, Department of Biochemistry, Cambridge, UK
^7^Computational and Systems Biology, Massachusetts Institute of Technology, Cambridge, MA, USA
^9^CNB-CSIC, Biocomputing Unit - Instruct Image Processing Center, Madrid, Spain
^10^Imperial College London, London, UK
^11^Weizmann Institute of Science, Rehovot, Israel
^12^Fox Chase Cancer Center, Institute for Cancer Research, Philadelphia, PA, USA
^13^Instituto de Ciencias de la Vid y del Vino (CSIC - Universidad de La Rioja - Gobierno de La Rioja), Oenology, Barcelona & Logroño, Spain
^14^Randall Centre for Cell & Molecular Biophysics, King's College London, London, UK
^15^European Molecular Biology Laboratory, Heidelberg, Germany
^16^Centre for Molecular Bioinformatics, Department of Biology, University of Rome Tor Vergata, Rome, Italy
^17^Charles University, Prague, Czech Republic
^18^University of Georgia, Department of Biochemistry and Molecular Biology & Institute of Bioinformatics, Athens, USA
^19^Indian Institute of Science Education and Research, Pune, India
^20^Ghent University, Department of Biomolecular Medicine, Ghent, Belgium
^21^VIB-UGent, Center for Medical Biotechnology, Ghent, Belgium
^22^University of Kent, Canterbury, Kent, UK
^23^Barcelona Supercomputing Center, Life Sciences Department, Barcelona, Spain
^24^University of Padova, Deptartment of Biomedical Sciences, Padova, Italy
^25^Centre for Genomic Regulation, Systems Biology, Barcelona, Spain
^26^Vrije Universiteit Brussel, Department of Bioengineering Sciences, Brussels, Belgium
^27^Facultad de Ingenieria, Universidad Nacional de Entre Rios, Oro Verde, Argentina
^28^University of Bergen, Department of Chemistry and Computational Biology Unit, Bergen, Norway
^29^University of Warsaw, Centre of New Technologies, Warsaw, Poland
^30^Masaryk University, CEITEC - Central European Institute of Technology and National Centre for Biomolecular Research, Faculty of Science, Brno, Czech Republic
^31^University College London, Department of Structural and Molecular Biology, London, UK
